# Aufbau von Interventionsdatenbanken für mehr Evidenzbasierung in Prävention und Gesundheitsförderung – methodische Überlegungen

**DOI:** 10.1007/s00103-021-03323-y

**Published:** 2021-04-09

**Authors:** Christin Rossmann, Annalena Bußkamp, Freia De Bock

**Affiliations:** Referat 2-22 „Zusammenarbeit mit Ländern, Krankenkassen und Verbänden, Gremien; Gesundes Alter; Frauengesundheit; Männergesundheit“, Bundeszentrale für gesundheitliche Aufklärung (BZgA), Maarweg 149–161, 50825 Köln, Deutschland

**Keywords:** Evidenzbasierte Maßnahmen, Kapazitätsentwicklung, Datenbanksystem, Wissenstransfer, Public Health, Evidence-based interventions, Capacity building, Database system, Knowledge transfer, Public health

## Abstract

Kommunale AkteurInnen erfahren bei der Entwicklung und Auswahl von Maßnahmen der Prävention und Gesundheitsförderung (MPG) keine ausreichende wissenschaftliche Unterstützung. Interventionsdatenbanken können Abhilfe leisten, indem sie wissenschaftlich abgesicherte und gut dokumentierte MPG präsentieren. Dadurch kann wissenschaftliche Evidenz zur Wirksamkeit einzelner Maßnahmen in lokale Entscheidungen einfließen. Die Datenbanken können ebenfalls dazu dienen, die Qualitätsentwicklung von Maßnahmen voranzutreiben.

In diesem Beitrag werden methodische und konzeptuelle Überlegungen erläutert, wie MPG für Datenbanken identifiziert und wie sie hinsichtlich ihrer Wirksamkeit eingeordnet und zielgruppengerecht aufbereitet werden können. Grundlage sind Erkenntnisse aus der Public-Health-Literatur und dem Projekt „Älter werden in Balance“ der Bundeszentrale für gesundheitliche Aufklärung (BZgA).

Systematische Übersichtsarbeiten sind für die Suche nach geeigneten MPG für die Datenbank nur bedingt geeignet, u. a. weil die darin aufgeführten Interventionstypen begrenzt und umsetzungsrelevante Angaben oft unvollständig dargestellt sind. Es erscheint aber vielversprechend, MPG aus der Praxis in die Datenbank einzustellen, wenn zusätzlich eine Einordnung hinsichtlich ihrer Wirksamkeit erfolgt bzw. Konsequenzen für die Weiterentwicklung und Evaluation formuliert werden. Es sollten zudem möglichst viele Informationen zur praktischen Umsetzung der MPG angegeben werden. In einem ersten Vorschlag wird gezeigt, wie eine Maßnahmenbeschreibung aussehen könnte. Zusätzlich sollten Manuale zur Durchführung von Maßnahmen zur Verfügung stehen.

**Zusatzmaterial online:**

Zusätzliche Informationen sind in der Online-Version dieses Artikels (10.1007/s00103-021-03323-y) enthalten.

## Abstract (en)

Municipal actors do not experience sufficient scientific support in the development and selection of preventive and health promoting interventions. Intervention databases can remedy this by presenting scientifically validated and well-documented interventions. This allows local decisions to be informed by scientific evidence on the effectiveness of interventions. The databases can also be used to promote quality development of interventions.

This paper explains methodological and conceptual considerations on how preventive and health-promoting interventions can be identified for databases and how they can be classified in terms of their effectiveness and prepared in a way that is appropriate for the target group. It is based on findings from the public health literature and the project “Ageing in Balance” (“Älter werden in Balance”) of the Bundeszentrale für gesundheitliche Aufklärung (BZgA).

Systematic reviews are of limited use in the search for suitable interventions for the database, partly because the types of interventions listed in them are limited and information relevant to implementation is often incompletely presented. However, it seems promising to include interventions from practice in the database if they are additionally classified with regard to their effectiveness or if consequences for further development and evaluation are formulated. In addition, as much information as possible should be provided on the practical implementation of interventions. A first suggestion shows what a description of the measures could look like. In addition, manuals for the implementation of measures should be available.

## Hintergrund

Prävention und Gesundheitsförderung sind laut Weltgesundheitsorganisation (WHO; [1]) 2 essenzielle Kernfunktionen bei der Förderung der öffentlichen Gesundheit. Kommunen haben im Sinne des Selbstverwaltungsrechtes und der Daseinsvorsorge eine steuernde Funktion und die politische Gestaltungskompetenz zur Weiterentwicklung und Schaffung präventiver und gesundheitsförderlicher Rahmenbedingungen. Die Kommunen sind dabei zentrale Akteure, da sie direkt in den Lebenswelten der BürgerInnen aktiv werden (z. B. Kindertagesstätten, Schulen, Sportvereine). Die lokale Gesundheitsförderung und Präventionsarbeit werden dabei meist übergreifend von kommunalen Gesundheitsämtern in Zusammenarbeit mit weiteren Ämtern (z. B. Umwelt, Stadtentwicklung) verantwortet [2].

Entscheidungen, welche Maßnahmen der Prävention und Gesundheitsförderung (MPG) auf kommunaler Ebene durchgeführt werden sollen, werden maßgeblich durch Faktoren wie politisches Mandat, Verfügbarkeit finanzieller und personeller Ressourcen sowie Interessen und Werte von lokalen Interessengruppen beeinflusst [3, 4].

Wissenschaftliche Evidenz (d. h. in unserem Verständnis Evidenz in Form von Daten, einschließlich epidemiologischen Studien, systematischen Übersichtsarbeiten, Projektevaluationen und qualitativen Studien [5]) als weitere Entscheidungsgrundlage für MPG erhält in der Praxis steigenden Zuspruch. Durch den Einbezug wissenschaftlicher Evidenz in die Entwicklung oder die Auswahl von MPG wird ein effizienteres Präventionssystem erwartet, das längerfristig positive gesundheitliche Auswirkungen auf die Bevölkerung haben soll [6, 7].

Der Einbezug von wissenschaftlicher Evidenz (insbesondere Wirksamkeitsstudien) durch AkteurInnen (AK) in Praxis und Politik ist mit Herausforderungen verknüpft. In Deutschland werden für die Evidenzbasierung von Prävention und Gesundheitsförderung zu wenig zuverlässige Wirksamkeitsnachweise für MPG durchgeführt [8, 9]. Evaluationsvorhaben werden häufig nicht prioritär finanziert oder die Evaluationen beschränken sich aufgrund begrenzter Budgets bzw. fehlender Methodenkompetenz auf kurzfristige Prozessergebnisse oder Vorher-nachher-Vergleiche von Zielparametern. Neben fehlendem Wissen in Praxis und Politik zur Abhängigkeit der Möglichkeit kausaler Aussagen vom Studiendesign ist diese Entwicklung möglicherweise auch auf kurzfristig ausgelegte politische Interessen zurückzuführen, z. B. den Wunsch nach Ergebnissen innerhalb einer Wahlperiode [3]. Das Präventionsgesetz von 2015 (inkl. weiterer Reformierung) und die in diesem Rahmen aufgesetzten Förderprogramme und Anforderungen könnten Qualitäts- und Wirksamkeitsanforderungen bei MPG erhöhen und damit Evidenzbasierung stärken [10].

Eine weitere Herausforderung ist, dass Entscheidungen zur Umsetzung von MPG auf lokaler Ebene in der Regel von AK in Praxis und Politik getroffen werden, deren Hauptaufgabe und -expertise nicht in der wissenschaftlichen Beurteilung der Wirksamkeit oder der Evaluationsergebnisse von Maßnahmen liegt. Hier fehlt eine strukturelle Unterstützung, mit der die MPG in einem Prozess für die kommunalen AK identifiziert, hinsichtlich der Wirksamkeit beurteilt und verständlich dargestellt werden [11].

Hier können Interventionsdatenbanken (im Folgenden „Datenbanken“ genannt), die wissenschaftlich abgesicherte, systematisch bewertete und gut dokumentierte MPG präsentieren, einen zentralisierten Zugang zu zusammengefasster Evidenz sowie Informationen zu effektiven MPG liefern [12] und so die Möglichkeit eröffnen, wissenschaftliche Evidenz zur Wirksamkeit einzelner Maßnahmen in lokale Entscheidungen einfließen zu lassen [11].

Mit Blick auf eine Verbesserung der Evidenzlage mit mehr zuverlässigen Wirksamkeitsnachweisen von MPG in Deutschland können Datenbanken auch als Instrument für den Einstieg in einen Qualitätsentwicklungsprozess genutzt werden [13]. Bestehende Angebote zur Beratung, Qualifizierung und Vernetzung für AK z. B. des Bündnisses der Gesetzlichen Krankenversicherungen (GKV; [14]) können, im Sinne der Kapazitätsentwicklung für eine evidenzbasierte Gesundheitsförderung, ergänzend wirken [11].

Bei der Entwicklung und dem Aufbau von Datenbanken müssen Entscheidungen in Bezug auf vorrangig 3 methodische Fragen getroffen werden:

1. Wie werden evidenzbasierte MPG für die Aufnahme in Datenbanken identifiziert?
2. Wie werden MPG hinsichtlich ihres Wirksamkeitsgrades klassifiziert?
3. Wie werden MPG für die Zielgruppe aufbereitet?

Der vorliegende Artikel gibt zu diesen 3 Fragen einen aktuellen Überblick über methodische und konzeptuelle Überlegungen aus der Public-Health-Wissenschaft und -Literatur und illustriert diese mit praktischen Erfahrungen in Bezug auf Methodik und Vorgehensweise beim Aufbau einer Datenbank im Rahmen des Projekts „Älter werden in Balance“ (ÄwiB) der Bundeszentrale für gesundheitliche Aufklärung (BZgA). Durch den wissenschaftlichen Überblick und die praktischen Erfahrungen und Abwägungen soll der vorliegende Übersichtsartikel dazu beitragen, das Verständnis der Vor- und Nachteile bestimmter Vorgehensweisen beim Aufbau solcher Datenbanken zu verbessern und diese als Instrumente zur Förderung der Evidenzbasierung in der Prävention und Gesundheitsförderung anzuerkennen.

## Identifizierung von evidenzbasierten Maßnahmen

**Figure 1:**
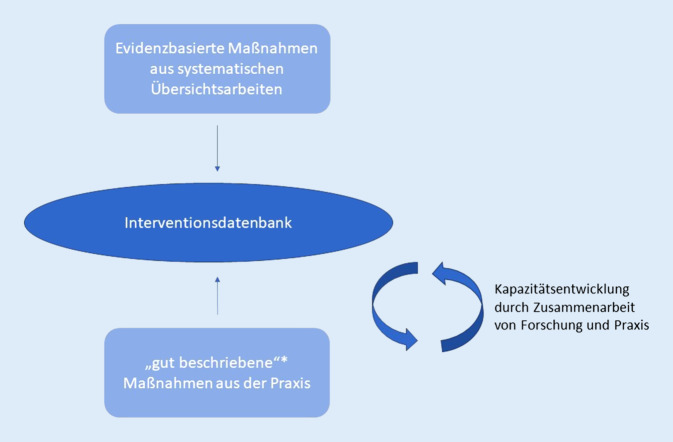

Konzeptuell lassen sich auf Basis einer internationalen Literaturrecherche zum Thema 2 Möglichkeiten ableiten, wie MPG für Datenbanken identifiziert werden können (Abb. 1):

Eine Möglichkeit ist die Identifizierung von evidenzbasierten MPG über die Erstellung von systematischen Übersichtsarbeiten [15], welche explizite, systematische Methoden nutzen, die mit Blick auf die Minimierung von Verzerrungen ausgewählt werden und so zuverlässige Erkenntnisse liefern. Sie können sowohl der Identifizierung von Einzelmaßnahmen als auch der Entwicklung von Leitlinien und Handlungsempfehlungen dienen (u. a. NICE Guidelines des National Institute for Care and Excellence; [16]). In beiden Anwendungsbereichen wird einem strukturierten, systematischen und reflektierten Prozess gefolgt. Methodik und Ergebnisse sind transparent und nachvollziehbar.

Eine andere Möglichkeit zur Identifizierung konkreter MPG ist das Einreichen von MPG aus der Praxis [17]. Mithilfe von Anreizen wie Preisen, Auszeichnungen und der Bekanntmachung wird für die Aufnahme in Datenbanken geworben. Die meisten MPG in bereits bestehenden Datenbanken in Deutschland (z. B. Praxisdatenbank des Kooperationsverbundes Gesundheitliche Chancengleichheit, Landeszentrum für Gesundheit NRW) stammen überwiegend aus der Praxis (und nicht der Wissenschaft) und wurden auch so eingereicht. Einige dieser Maßnahmen können als „evidenzbasiert“ gelten, andere haben das Potenzial, in Richtung Evidenzbasierung weiterentwickelt zu werden.

### Maßnahmen aus systematischen Übersichtsarbeiten

Insbesondere in Bezug auf MPG im deutschsprachigen Raum, aber auch international ist bei der Identifizierung von MPG über systematische Übersichtsarbeiten problematisch, dass durch die derzeitige Evidenzlage im Feld der Prävention und Gesundheitsförderung – insbesondere bei einer Fokussierung auf MPG mit nachgewiesener Wirksamkeit und dafür geeigneten Studiendesigns [18] – nur recht eng begrenzte Interventionstypen (u. a. vor allem verhaltenspräventive Programme für ausgesuchte Zielgruppen), Outcomes und Resultate abgedeckt werden. Diese entsprechen oftmals nicht der komplexen Natur von vielen MPG (z. B. verhältnispräventive Maßnahmen, komplexe Maßnahmen in Lebenswelten). Die Komplexität und Heterogenität dieser Maßnahmen erschweren die Vergleichbarkeit untereinander, sodass quantitative Metaanalysen der Effekte kaum möglich sind [19]. Die narrative Synthese in systematischen Übersichtsarbeiten ist daher die am häufigsten genutzte Analyseform in Public-Health-Übersichtsarbeiten. Sie ist jedoch maßgeblich abhängig von dem Verfassenden der Übersichtsarbeit und aufgrund der subjektiven Interpretation anfällig für Verzerrungen [20].

Aus diesem Grund sowie aus dem Bedarf an möglichst schnell verfügbaren Übersichtsarbeiten für Politik und Praxis haben sich in den vergangenen 10–15 Jahren alternative Methoden zu konventionellen Übersichtsarbeiten entwickelt [21]. Beispielsweise soll in einem Realist-Review beantwortet werden, wie und warum komplexe Interventionen in bestimmten Situationen funktionieren, anstatt davon auszugehen, dass sie entweder funktionieren oder nicht [22]. Dadurch wird ein detailliertes Bild der Mechanismen komplexer Interventionen vermittelt, das auch kommunale AK bei der Planung und Umsetzung von Maßnahmen unterstützen könnte [19–22].

### Erfahrungen mit systematischen Literaturrecherchen im Projekt ÄwiB

Für die Identifizierung von evidenzbasierten MPG für die Aufnahme in die ÄwiB-Datenbank wurde zunächst eine systematische Literaturrecherche als Methode gewählt. Mit spezifischen Schlüsselwörtern und datenbankspezifischen MeSH (Medical Subject Headings) wurde in den Datenbanken Pubmed, CINAHL und Livivo systematisch nach evidenzbasierten Maßnahmen (Kontrollgruppe oder -situation als Einschlusskriterium) recherchiert. Insgesamt wurden 203 Artikel identifiziert und schlussendlich 26 Artikel in die qualitative Analyse eingeschlossen.

In diesen 26 Artikeln zu MPG waren jedoch nur wenige Informationen enthalten, die sich auf die Umsetzung der jeweiligen Maßnahme bezogen. Bei allen 26 Artikeln fehlten z. B. Informationen zu personeller Ausstattung, finanziellem Rahmen und/oder Implementierungsschritten. Nur bei 14 von 20 Artikeln konnten förderliche und hinderliche Faktoren bei der Implementierung und Umsetzung aus den Beschreibungen im Diskussionsteil des Artikels entnommen werden. Diese bezogen sich jedoch größtenteils auf die Umsetzung der Evaluationsstudie selbst als auf die eigentliche Umsetzung der MPG. Lediglich die Zielgruppe wurde in allen Artikeln definiert. Zusätzliche praxisrelevante Anlagen (z. B. detaillierte Beschreibung der Intervention) wurden nur in 3 von 26 Artikeln zur Verfügung gestellt.

Da bei dieser systematischen Recherche nur nach Studien mit Wirksamkeitsnachweis recherchiert wurde, waren möglicherweise Angaben zur Implementierung, Machbarkeit („feasibility“) und Kosteneffektivität aus Sicht der AutorInnen nicht prioritär. Die Ergebnisse der systematischen Recherche in ÄwiB zeigen jedoch, dass die Beschreibungen der MPG den Informationsbedürfnissen von kommunalen AK nicht gerecht werden. Aus diesem Grund wäre es sinnvoll, in wissenschaftlichen Publikationen zu MPG Manuale mit umsetzungsrelevanten Angaben als Anhang zur Verfügung stellen.

### Maßnahmen aus der Praxis

MPG, die aus der Praxis stammen, unterliegen einem Selektionsbias, da sie von AK eingereicht werden [24]. Trotzdem birgt der Ansatz wichtige Vorteile, die das Potenzial einer Datenbank vergrößern können. Neben der Listung und Bewertung von Maßnahmen kann die Datenbank als Instrument für den Einstieg in einen Qualitätsentwicklungsprozess genutzt werden [13]. Auf Grundlage bestimmter Aufnahme- und Bewertungskriterien kann ein Qualitätsstandard definiert werden. Somit könnten im Zeitverlauf immer mehr qualitativ hochwertige, evidenzbasierte MPG zur Dissemination zur Verfügung stehen [13, 27].

Durch eine aktive Zusammenarbeit zwischen den Verantwortlichen für die (wissenschaftsgeleitete) Datenbank und den InitiatorInnen der MPG aus der Praxis, z. B. bei der Bekanntmachung bzw. beim Hochladen/Einreichen der Projekte, kann ein Qualitätsentwicklungsprozess angestoßen werden, der in dieser Art bei Aufnahme von MPG durch systematische Literaturrecherchen nicht möglich ist. Bei bereits wissenschaftlich publizierten Ergebnissen sind die MPG oft bereits beendet, AnsprechpartnerInnen nicht mehr vorhanden oder international, sodass kein auf das deutsche Feld der Prävention und Gesundheitsförderung gerichteter Qualitätsentwicklungsprozess möglich ist.

Es können bestimmte Aufnahmekriterien für MPG formuliert werden, wie etwa eine „gute Beschreibung“ der Maßnahmen (vgl. unten: Spektrum der wissenschaftlichen Absicherung). Der weitere Prozess könnte durch wissenschaftliche Institutionen begleitet werden, die bei der Konzeption mit wissenschaftlichen Erkenntnissen, Ziel- und Maßnahmenformulierungen sowie der Definition von Outcomes die (Weiter‑)Entwicklung unterstützen und Voraussetzungen für eine Evaluation schaffen. Gleichzeitig gäbe es Raum für gegenseitigen Austausch von Erfahrungswissen, kontextspezifischem Wissen, Voraussetzungen (finanziell, personell), persönlichen Präferenzen und Werten aus der Praxis. Eine frühzeitige Zusammenarbeit zwischen Praxis und Forschung innerhalb dieses Prozesses kann helfen, Fragen der Auswahl von relevanten Maßnahmen auf Basis einer strukturierten Bedarfs- und Bedürfnisanalyse im Zielkontext [25] besser in die Entwicklung der MPG einzubeziehen. Dies betrifft auch Anforderungen von Informationen zu MPG in Bezug auf Implementierung, Umsetzung und die evaluatorischen Anforderungen [26].

## Klassifizierung von Maßnahmen nach ihrem Wirksamkeitsgrad

In der Prävention und Gesundheitsförderung ist es oftmals notwendig zu handeln, ohne dass eine ausreichende Evidenzbasierung der entsprechenden Maßnahme vorliegt. Obwohl mit Recht gefordert wird, dass Wirksamkeitsnachweise für MPG erbracht werden müssen, muss bei der Klassifizierung auch berücksichtigt werden, dass MPG trotz eines noch fehlenden Nachweises wirksam sein können. Dies kann z. B. über die Einordnung von MPG in einem „Spektrum der wissenschaftlichen Absicherung“ innerhalb der Datenbank erreicht werden, das transparent kennzeichnet, inwieweit eine MPG wissenschaftlich bereits abgesichert ist bzw. welche konkreten Anforderungen für die weitere Evaluation und Implementierung abgeleitet werden können [11].

Um MPG mit geringer wissenschaftlicher Absicherung, aber möglicherweise hohem Wirkungspotenzial nicht auszuschließen, erstellten Veerman and van Yperen [28] im Jahr 2007 ein Entwicklungsmodell, das MPG nach ihrer Wirksamkeit von „potenziell effektiv“ bis „wirksam“ einordnet. Auf Grundlage dieser anfänglichen Überlegungen zu Evidenzstufen in Prävention und Gesundheitsförderung sowie weiterführender Überlegungen u. a. durch Flay et al. [29] und Gottfredson [30] wird im Memorandum der BZgA [11] für Deutschland ein dreistufiges Spektrum der wissenschaftlichen Absicherung vorgeschlagen:

1. Praxisprojekte: „gut beschriebene“ MPG, die eine Beschreibung der essenziellen Elemente wie Ziele, Zielgruppe, Methoden, Aktivitäten und Voraussetzungen enthalten,
2. BZgA Promising Practice („vielversprechende“ Praxis): MPG mit plausiblen Wirksamkeitsvoraussetzungen (z. B. aufgrund eines theoretisch oder empirisch abgesicherten Wirkmodells), aber ohne Ergebnis einer Effektevaluation,
3. BZgA Best Evidence (beste Evidenz): MPG, die einen kausalen Wirksamkeitsnachweis vorweisen können.

Die Einordnung von MPG ist der notwendige erste Schritt, um darauffolgend konkrete Anforderungen für die Implementierung und Evaluation zu formulieren. Durch Zusammenarbeit von Forschung und Praxis im Qualitätsentwicklungsprozess sowie finanzielle Anreize [15] kann Kapazität entwickelt werden, wie es zum Beispiel in den Niederlanden (am Rijksinstituut voor Volksgezondheid en Milieu, RIVM) durchgeführt wird. Dort wird durch die finanzielle Unterstützung des niederländischen Ministeriums für Gesundheit, Wohlbefinden und Sport sowie den lokalen Gesundheitsbehörden die Auflistung von anerkannten MPG in Datenbanken gefördert. Für die praktische Umsetzung werden Onlineinformationen zu Interventionen, Evidenzzusammenfassungen sowie Instrumente zur Auswahl der Interventionen geliefert. Mit weiterbildenden Workshops zur Auswahl von Interventionen sowie dem festgelegten Austausch zwischen Forschung und Praxis wird Kapazität im Bereich Prävention und Gesundheitsförderung entwickelt [31].

### Erfahrungen bei der Einordnung von Praxismaßnahmen nach ihrem Wirksamkeitsgrad im Projekt ÄwiB

Neben den MPG aus der systematischen Literaturrecherche wurde beim Aufbau der Datenbank für ÄwiB auch auf Projekte aus der Praxis zurückgegriffen. Im Zeitraum August bis September 2019 wurden in insgesamt 6 Datenbanken bewegungsförderliche Maßnahmen für ältere Menschen mithilfe des jeweils datenbankspezifischen Suchmechanismus recherchiert (Praxisdatenbank des Kooperationsverbunds Gesundheitliche Chancengleichheit, Praxisdatenbank „Gesund und aktiv älter werden“ der BZgA, IN FORM Projektdatenbank, Datenbank SPOFOR, Projektdatenbank des Landeszentrums Gesundheit NRW, Projekte auf der Website des Deutschen Turnerbunds). Eingeschlossen wurden Maßnahmen, die für ältere Menschen bestimmt sind, Bewegung fördern sowie in der Kommune durchgeführt werden können. Insgesamt wurden 125 Projekte identifiziert.

Alle Projekte wurden in das „Spektrum der wissenschaftlichen Absicherung“ eingeordnet. Zuerst wurden die Beschreibungen auf das Vorhandensein der gewünschten Informationen aus Kategorie 1 („Praxisprojekt“) geprüft. Waren die Informationen nicht vollständig, wurde durch eine einfache Google-Recherche versucht, die fehlenden Informationen zu ergänzen. Im zweiten Schritt wurden die Projekte über die Projektbeschreibungen selbst, weitere Abschluss- und Evaluationsberichte oder Wirksamkeitsstudien auf Vorhandensein der Informationen aus Kategorie 2 („BZgA Promising Practice“) und 3 („BZgA Best Evidence“) geprüft.

**Figure 2:**
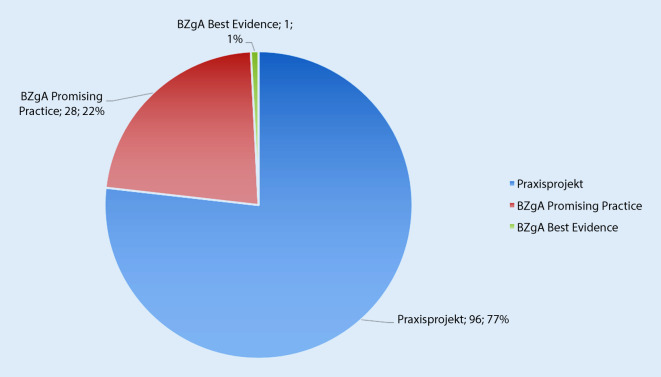

Die Einordnung führte zu 96 Projekten in der Kategorie „Praxisprojekt“, 28 Projekten in der Kategorie „BZgA Promising Practice“ und 1 Projekt in der Kategorie „BZgA Best Evidence“ (Abb. 2). Von 96 „Praxisprojekten“ wurden 15 Projekte vorerst zurückgestellt, da die Informationen zwar vorhanden waren, jedoch die Qualität der Beschreibung eher unzureichend für die Einordnung in die Kategorie „Praxisprojekt“ war.

## Aufbereitung und Beschreibung der Maßnahmen für die Zielgruppe

Bei der Auswahl von relevanten MPG müssen die NutzerInnen der Datenbank erkennen können, ob für die ermittelten Bedarfe und Bedürfnisse ihrer jeweiligen Zielgruppe effektive MPG vorhanden sind (und wie effizient diese im Vergleich zu alternativen oder bereits bestehenden MPG sind; [32]). Interventionen, bei denen die Zielgruppe und der Kontext am ähnlichsten sind, sollten bei der Suche in die engere Wahl gezogen werden [33]. Dazu müssen MPG aber zumindest „gut beschrieben“ sein (siehe „Praxisprojekte“ oben). Zusätzlich sollten z. B. eine Beschreibung des Kontextes und umsetzungsrelevante Informationen enthalten sein (z. B. Implementierungsschritte, finanzieller Rahmen, personelle Ressourcen).

Die Studienlage dazu, wie MPG für eine erfolgreiche Umsetzung in der Praxis beschrieben werden sollten, ist derzeit noch unzureichend. Indirekt können jedoch Studien, die allgemeine Informationsbedürfnisse von verschiedenen StakeholderInnen in Prävention und Gesundheitsförderung analysiert haben, hilfreiche Hinweise für die Projekt- und Maßnahmenbeschreibung liefern. Barr-Walker [34] fand in seiner Übersichtsarbeit heraus, dass Mitarbeitende des Gesundheitsamts vor allem „praktisches Wissen“ zur Umsetzung von Maßnahmen benötigen, führte diesen Bedarf jedoch nicht weiter aus. Bei der Entscheidungsfindung sind laut Satterfield [35] neben wissenschaftlicher Evidenz auch Informationen zu Ressourcen (finanziell, personell), Populations- und Zielgruppencharakteristika, den Präferenzen und Werten der Zielgruppe sowie zur Expertise der AK notwendig. Für die interventionsbezogene Wissenschaft sind Checklisten vorhanden, die bei wissenschaftlichen Publikationen einen gewissen Reportingstandard unterstützen und die teils auch von wissenschaftlichen Fachzeitschriften als Voraussetzung für die Annahme einer wissenschaftlichen Publikation definiert werden. Diese Checklisten schlagen vor, wie Interventionen beschrieben werden können, damit andere sie umsetzen und replizieren können (u. a. TIDieR „Template for Intervention Description and Replication“ und StaRI „Standards of Reporting Implementation Studies“ für die Berichterstattung von Implementierungsstudien; [30, 36, 37]). Diese Tools dienen der Verbesserung der Qualität bei der Berichterstattung von Implementierungsstudien für die Forschungsgemeinschaft, jedoch wurden sie in ihrer Anwendung bisher nicht für AK der kommunalen Politik und Praxis in Deutschland getestet. Ein Reportingstandard für MPG aus der Praxis existiert nach unserem Kenntnisstand nicht. Ein erster Aufschlag eines spezifischen Formats für MPG-Projektbeschreibungen wurde im Rahmen von ÄwiB erarbeitet (siehe unten).

Nach Auswahl von geeigneten Maßnahmen aus einer Datenbank durch AK aus der Praxis (ggf. mithilfe von Entscheidungsinstrumenten) wäre abzuschätzen, ob und wie die jeweilige Maßnahme in den Zielkontext übertragbar ist (Transferabilität; [38, 39]; siehe auch Beitrag von Schloemer et al. in diesem Themenheft) und damit die gleiche Wirksamkeit wie im Originalkontext zu erwarten ist. Hierzu könnten Datenbanken in Zukunft auch Kontakte und Austausch zwischen AnsprechpartnerInnen des Originalprojektes und AK ermöglichen.

### Erfahrungen bei der Beschreibung von Maßnahmen im Projekt ÄwiB

Eine im Rahmen von ÄwiB angelegte Studie zur Translation von wissenschaftlichen Erkenntnissen ergab, dass Informationen zu Übertragbarkeit, Setting, Rahmenbedingungen, Kosten sowie förderlichen und hinderlichen Faktoren hilfreich für kommunale AK sind (siehe auch Beitrag von Bußkamp et al. in diesem Themenheft). Auf Grundlage dieser Ergebnisse wurde ein Vorschlag für eine Projektbeschreibung erarbeitet, der zur Implementierung von „gut beschriebenen“ MPG (siehe „Praxisprojekte“ oben) in Datenbanken geeignet ist. Die Struktur der Projektbeschreibung wurde so gestaltet, dass nach der eigentlichen Beschreibung der MPG, Hinweise für die Umsetzung der Intervention gegeben werden können. In Infobox 1 ist die inhaltliche Struktur dieser Projektbeschreibung dargestellt, ein Beispiel befindet sich im Onlinematerial zu diesem Beitrag.

Die recherchierten Praxisprojekte wurden anhand des entwickelten Formats neu für die Datenbank in ÄwiB aufbereitet. Da hier – zwar in geringerem Maße als bei der systematischen Recherche – aber auch Informationen zur Umsetzung fehlten, die nicht durch eine Internetrecherche ergänzt werden konnten, werden diese aktuell bei den genannten Kontaktpersonen (*n* = 125) angefragt. Von bisher 54 kontaktierten Personen haben sich 20 nach einer vierwöchigen Zeitspanne zurückgemeldet (Stand Januar 2021). Zwar wurde durch die Ergänzungen der Kontaktpersonen in den Steckbriefen deutlich, dass die Vollständigkeit und Praxisrelevanz der Beschreibungen maßgeblich von der Zusammenarbeit zwischen den Verantwortlichen der Datenbank sowie der Kontaktpersonen der Projekte abhängig sind, jedoch bedeutet diese Informationsbeschaffung im Nachhinein einen hohen Personal- und Zeitaufwand. Bezogen auf die bisherige Rücklaufquote ist dies ein wenig effizientes Vorgehen und könnte durch ein im Vorhinein festgelegtes Beschreibungsformat für die Datenbank ersetzt werden.

## Fazit

Zur Förderung von Evidenzbasierung und Qualität in Prävention und Gesundheitsförderung in Deutschland können Interventionsdatenbanken einen entscheidenden Beitrag leisten. Die Methodik bei Entwicklung und Aufbau einer Datenbank bestimmt dabei die Funktion sowie die Möglichkeit, inwiefern Datenbanken auch den Einstieg in einen Qualitätsentwicklungsprozess im Sinne von Kapazitätsentwicklung bei AK in Praxis und Politik ermöglichen. Im Rahmen dieses Artikels wurden auf Basis wissenschaftlicher Literatur in Public Health und des Diskurses im Feld der Prävention und Gesundheitsförderung Vorgehensweisen und Möglichkeiten präsentiert, die Anforderungen und mögliche Funktionen von Datenbanken bei der Förderung von Evidenzbasierung und Qualität verdeutlichen. Diese Möglichkeiten wurden durch Erfahrungen und Prozessdaten beim Aufbau einer Datenbank mit Maßnahmen der Prävention und Gesundheitsförderung (MPG) aus dem Projekt ÄwiB illustriert.

Zu Beginn des Aufbaus einer Datenbank muss überlegt werden, welcher Ansatz zur Aufnahme von MPG in die Datenbank verwendet werden soll. Nach unserer Auffassung empfiehlt sich die Aufnahme von MPG in eine Datenbank durch Einreichung aus der Praxis vor allem dann, wenn gleichzeitig Ressourcen für Qualitätsentwicklungsprozesse vorhanden sind. Zusätzlich wäre eine finanzielle Förderung von Wirksamkeitsevaluationen bei vielversprechenden MPG mit noch ausstehendem Wirksamkeitsnachweis sinnvoll. Damit könnten mehr Maßnahmen mit zuverlässigem Wirksamkeitsnachweis in Datenbanken zur Verfügung stehen und die Evidenzbasierung in Prävention und Gesundheitsförderung gestärkt werden.

Um bei der Förderung von Evidenzbasierung auch Signale in Praxis und Politik zu setzen, sollten MPG in Datenbanken hinsichtlich des Nachweises von Wirksamkeit transparent kategorisiert werden. Werden MPG ausgehend von einem Entwicklungsmodell betrachtet, können durch diese Transparenz auch Konsequenzen für eine Weiterentwicklung klar formuliert werden. Die Konsequenzen beziehen sich dabei nicht alleine auf die Erbringung eines Nachweises der Wirksamkeit, sondern insbesondere auf die konzeptionelle Ausarbeitung von logischen Modellen oder Wirkmodellen, die das Wirkungspotenzial von MPG darlegen können („BZgA Promising Practice“).

Um kommunalen AK die Auswahl an bedarfs- und bedürfnisorientierten MPG zu erleichtern, sollten MPG in Datenbanken verständlich aufbereitet zur Verfügung stehen. Das bedeutet auch, dass Informationen zur praktischen Umsetzung und Übertragbarkeit vorliegen sollten. Auf Basis der Literatur und unserer praktischen Erfahrungen im Rahmen von ÄwiB schlagen wir ein erstes, möglichst kurzes und trotzdem bedarfsgerechtes Reportingformat für MPG vor. Allerdings zeigen unsere Erfahrungen auch, dass die in diesem Reportingformat erforderlichen Angaben selten befriedigend gemacht werden können. Tatsächlich fehlen relevante Informationen aus wissenschaftlichen Publikationen zu MPG, die für die NutzerInnen hoch relevant wären. Daher sollten wissenschaftliche Publikationen zu MPG in Zukunft verpflichtend von Manualen begleitet sein, die z. B. als Anhang zur Verfügung gestellt werden könnten und die mindestens die von uns definierten Kriterien des Reportingformats erfüllen sollten.

### Infobox 1 Inhaltliche Struktur einer Projekt- und Maßnahmenbeschreibung zur Aufnahme von Maßnahmen der Prävention und Gesundheitsförderung in Interventionsdatenbanken. Vorschlag aus dem Projekt ÄwiB

*1. Beschreibung*

- Ziel(e)
- Projektzeitraum
- Maßnahmen/Arbeitsprogramm
- (Geplante) Ergebnisse
- Hinweise zur Wirksamkeit
- Kontakt
- Nähere Informationen (z. B. Projektwebsite)
- Kooperation(en)
- Sponsor(en)

*2. Hinweise zur Umsetzung*

- Zielgruppe
- Teilnahmevoraussetzungen
- Empfehlungen zur personellen Ausstattung
- Finanzieller Rahmen
- Empfohlene Implementierungsschritte
- Erfolgsfaktoren und Stolpersteine
- Anlage(n) (z. B. Abschlussberichte, Praxismanuale)

## Supplementary Information


