# Supplementary material for: Development of intervention databases to promote evidence-based prevention and health promotion—methodological considerations
Source: Bundesgesundheitsblatt Gesundheitsforschung Gesundheitsschutz. 2021 Apr 9;64(5):544–51. [Article in German] doi: 10.1007/s00103-021-03323-y (PMC8087547; doi:10.1007/s00103-021-03323-y)
Supplement: Supplementary file 1 [file 103_2021_3323_MOESM1_ESM.pdf]

Bundesgesundheitsblatt 5/2021

Elektronisches Zusatzmaterial zum Beitrag:

Aufbau von Interventionsdatenbanken für mehr Evidenzbasierung in Prävention und Gesundheitsförderung – methodische Überlegungen

Christin Roßmann[1], Annalena Bußkamp[1], Freia De Bock[1]

[1]Bundeszentrale für gesundheitliche Aufklärung (BZgA), Abteilung 2, Köln, Deutschland

Korrespondenzadresse:

Christin Roßmann
Referat 2-22 „Zusammenarbeit mit Ländern,
Krankenkassen und Verbänden, Gremien;
Gesundes Alter; Frauengesundheit; Männergesundheit“
Bundeszentrale für gesundheitliche Aufklärung (BZgA)
Maarweg 149-161
50825 Köln, Deutschland
christin.rossmann@bzga.de

Inhalt:

Beispiel einer Projektbeschreibung anhand des im Projekt „Älter werden in Balance“ (ÄwiB) entwickelten Formats zur Aufbereitung von Maßnahmen

Beispiel einer Projektbeschreibung anhand des im Projekt „Älter werden in Balance“ (ÄwiB) entwickelten Formats zur Aufbereitung von Maßnahmen

Das AlltagTrainingsProgramm (ATP)

Das ATP ist ein Bewegungsangebot für Menschen ab 60 Jahren und Baustein des BZgA-Präventionsprogramms „Älter werden in Balance“, das ältere Menschen darin unterstützt, ihre Gesundheit und ihr Wohlbefinden durch mehr körperliche Aktivität im Alltag zu erhalten.

Über das Projekt

**Ziel(e)**	Das ATP will einen Beitrag dazu leisten, Rahmenbedingungen für eine Verbesserung der Gesundheit und des Wohlbefindens der älter werdenden Bevölkerung zu schaffen (Verhältnisprävention).<br><br>Als Maßnahme der individuellen Gesundheitsförderung will das ATP zudem eine dauerhafte Verhaltensveränderung bei den Menschen in ihrem Alltag bewirken (Verhaltensprävention).<br><br>Ziele des ATP:<br><br>• Möglichst viele – vor allem inaktive – Menschen ab 60 Jahren in Bewegung bringen und dadurch zu einem aktiven Lebensstil dieser Altersgruppe beitragen.<br>• Den Alltag älterer Menschen drinnen und draußen als Trainingsmöglichkeit verstehen und nachhaltig aktiver gestalten.<br>• Ältere Menschen dabei unterstützen, die für ihren Alltag wichtigen Fähigkeiten wie Kraft, Ausdauer, Koordination<br>und Beweglichkeit möglichst alltagsnah zu trainieren.
**Projektzeitraum**	Entwicklung: 2014<br>Start: 2016 (fortlaufend)
**Maßnahmen/Arbeitsprogramm**	Das ATP gibt es in zwei Varianten:<br><br>**ATP-Präventionskurs**<br>(ATP – Ganzkörperkräftigungsprogramm mit Alltagsbezug)<br><br>Das Einstiegsangebot richtet sich an inaktive Menschen ab 60 Jahren. Der Kurs findet über 12 Wochen mit jeweils einer einstündigen Kurseinheit pro Woche statt. Jede der 12 Kursstunden behandelt ein spezielles Thema mit Bezug zum Alltag.<br>Das ATP ist in dieser Form von der Zentralen Prüfstelle Prävention (ZPP) als Präventionskurs anerkannt und mit dem Siegel „Deutscher Standard Prävention“ zertifiziert. Wird das ATP als Präventionskurs angeboten, kann es von den Krankenkassen bezuschusst werden.

	**ATP-Dauerangebot**<br>(Alltag in Bewegung – das Dauerangebot zum ATP)<br><br>Das ATP-Dauerangebot wurde auf Basis des ATP-Präventionskurses weiterentwickelt, um älteren Menschen ein zeitlich unbegrenztes, fortlaufendes Angebot anzubieten. Es dient der nachhaltigen Bewegungsförderung, um eine langfristige Bindung an einen aktiven Lebensstil zu vermitteln.<br>Den Übungsleitenden steht ein Übungspool zur Verfügung, aus dem sie einzelne Übungen auswählen und für die jeweilige Übungseinheit zusammenstellen können.<br><br>Für beide ATP-Varianten gilt: Das Training kann in Alltagsbekleidung und ohne den Einsatz von Fitnessgeräten umgesetzt werden. Die Teilnehmenden bekommen gezeigt, wie sie alltägliche Bewegungen (z.B. Gehen, Aufstehen, Hinsetzen, Tragen, Überkopfheben, Strecken, Ziehen) gezielt trainingswirksam nutzen und ihre Umgebung in das Training einbeziehen können. So wird die Treppe zum „Fitnessgerät“, um Ausdauer, Kraft und Koordination zu verbessern.
**Ergebnisse**	Das ATP wurde in der Pilotphase 2016 beim Landessportbund NRW durchgeführt und von der Deutschen Sporthochschule sowie der Hochschule Magdeburg-Stendal evaluiert.<br><br>Der ATP-Präventionskurs wurde hinsichtlich Gruppengröße und Kurszusammensetzung auf einer Schulnotenskala durchschnittlich mit gut+ bewertet. 94,5% der befragten Teilnehmenden gaben an, wieder am ATP teilnehmen zu wollen und 100% würden das ATP weiterempfehlen. Des Weiteren geben zwei Drittel an, dass sie drei Monate nach Ende des ATP-Kurses noch immer Kontakt zu Personen aus der Trainingsgruppe oder zu einem Übungsleiter oder Übungsleiterin haben. 95% bzw. 93% der Teilnehmenden gelang es, direkt bzw. drei Monate nach der Kursteilnahme mehr Bewegung in ihren Alltag zu integrieren.<br><br>Die positiven Ergebnisse der Teilnehmenden-Befragung zur Akzeptanz des Kurses werden durch die Ergebnisse der Übungsleitenden- und Expertenbefragung gestützt. Eine Auswertung der Interviews zeigte, dass das ATP-Kursprogramm von den befragten Expertinnen und Experten einheitlich positiv und innovativ bewertet wurde.<br><br>Als besonders wirksam in Bezug auf die Akquise von Teilnehmenden haben sich die direkte persönliche Ansprache sowie die Bewerbung in Printmedien (z. B. Zeitungen) herausgestellt. Bei „vulnerablen“ Zielgruppen zeigte Vereinswerbung den größten Erfolg. Um als Umsetzende auf das ATP aufmerksam zu werden, bedarf es eines grundlegenden persönlichen Interesses an der Bewegungsvermittlung und speziell an der Seniorenarbeit. Das ATP wird dabei häufig als Erweiterung des Angebotsspektrums verstanden.
**Wirksamkeit**	✓ Erfüllt Aufnahmekriterien (Praxisprojekt)<br>✓ Vielversprechend (BZgA Promising Practice)<br>Nachgewiesen effektiv (BZgA Best Evidence)
**Kontakt**	Bundeszentrale für gesundheitliche Aufklärung<br>Ansprechperson: [Name Ansprechperson]<br>E-Mail: atp[at]bzga.de<br>Telefon: [Nummer]

Sie wollen diese Maßnahmen in Ihrer Gemeinde/Ihrem Stadtteil Ihrer Kommune umsetzen? Diese Informationen und Erfahrungen aus dem Projekt können Ihnen helfen.

**Zielgruppe**	Frauen und Männer ab 60 Jahren, die in den letzten Jahren körperlich eher inaktiv waren und daran etwas ändern möchten.<br>Im Hinblick auf das ATP-Dauerangebot alle älteren Menschen, die sich bereits sportlich betätigen und dauerhaft aktiv sein möchten.<br>Besondere Aufmerksamkeit gilt älteren Menschen, die seltener von Angeboten der Gesundheitsförderung erreicht werden, zugleich aber anfälliger für Krankheiten sind.
**Teilnahmevoraus-setzung**	Die körperliche Minimalvoraussetzung für die Teilnahme lautet, dass die Teilnehmenden ohne äußere Unterstützung (z.B. Hilfs-<br>mittel für das Gehen) ihren Alltagsaktivitäten nachgehen können. Mit Ausblick auf eine 60-minütige bewegungsaktive Einheit sollten diese zusätzlich in der Lage sein, 30 Minuten lang spazieren zu gehen.
**Empfehlungen zur personellen Ausstattung**	Qualifikationsanforderungen:<br>Zur Durchführung eines ATP-Präventionskurses müssen die Übungsleitenden eine staatlich anerkannte Bewegungsfachausbildung oder die 2. Lizenzstufe (ÜL-B) „Sport in der Prävention“ des DOSB-Lizenzsystems vorweisen. Außerdem müssen sie an einer ATP-Schulung für das Kursangebot teilgenommen haben.<br><br>Für das ATP-Dauerangebot benötigen die Übungsleitenden die 1. Lizenzstufe (ÜL-C), Erfahrungen im Umgang mit Älteren und müssen an einer ATP-Schulung für Kurs oder Dauerangebot teilgenommen haben.
**Finanzieller Rahmen**	Die Förderung der ATP-Kurse durch die BZgA zwischen 2016 und 2018 betrug 800,00 Euro pro Kurs. Zu den benötigen Kosten für das ATP-Dauerangebot können keine Angaben gemacht werden.
**Empfohlene Implementierungs-schritte**	Für Vereine:<br>Je nach ATP-Variante, benötigen Sie einen Übungsleitenden mit einer B- oder C-Lizenz, die/der eine ATP-Schulung absolvieren muss. Diese werden von den Landessportbünden oder Landesturnverbänden angeboten. Sie benötigen darüber hinaus eine freie Turnhalle. Zur Bewerbung des Angebots bieten sich Flyer, Aushänge, Pressemeldungen, Anzeigen sowie Informationen über die Kommunikationskanäle des Vereins an.<br><br>Für weitere Institutionen:<br>Wenn lokale Akteure, wie z.B. Kommunen, Wohlfahrtsverbände, Kirchengemeinden, Seniorenbeiräte, soziale Einrichtungen oder Wohnungsbaugesellschaften Kurse für ältere Menschen anbieten wollen, die eher nicht von Vereinsangeboten erreicht werden, braucht es ein breites Netzwerk verschiedener Partnerorganisationen. Hilfreich ist darüber hinaus eine feste Ansprechpartnerin/einen fester Ansprechpartner, die/der mit örtlichen Sportvereinen und anderen Interessengruppen zusammenarbeitet und vor diesem Hintergrund Schritte unternehmen kann, um einen Raum (das kann auch ein Pfarrsaal sein!) und einen Übungsleitenden zu finden sowie eine Finanzierung des Angebots sicherzustellen. Diese/dieser spricht über verschiedene Wege die Zielgruppe an und motiviert diese zur Teilnahme.
**Erfolgsfaktoren und Stolpersteine**	Erfolgsfaktoren:<br>• Einbindung des ATP in bestehende Vereinsstrukturen des organisierten Sports<br>• Zertifizierung durch Siegel „Sport pro Gesundheit“ (DOSB) und „Pluspunkt Gesundheit“ (DTB)

	• Anerkannter Präventionskurs im Programm der Zentralen Prüfstelle Prävention (ZPP) (Siegel „Deutscher Standard Prävention“)<br>• Niedrigschwellige Umsetzbarkeit vor Ort<br><br>Stolpersteine:<br>• Sicherung einer nachhaltigen Finanzierung<br>• Erreichbarkeit vulnerabler Zielgruppen
**Kooperation(en)**	Bundeszentrale für gesundheitliche Aufklärung<br>Deutsche Sporthochschule Köln<br>Deutscher Olympischer Sportbund e.V.<br>Deutscher Turner-Bund e.V.<br>Landessportbund Nordrhein-Westfalen e.V.
**Sponsor(en)**	Verband der Privaten Krankenversicherung e.V.
**Anlagen**	Die Materialien für Übungsleiterinnen und Übungsleiter können nur im Rahmen einer Schulung zur Verfügung gestellt werden.<br><br>Für interessierte Menschen aus der Zielgruppe oder Institutionen gibt es die „Bewegungspackung“ mit Übungen für „Zuhause“. Es handelt sich um eine kleine Schachtel. Darin enthalten sind neben dem Beipackzettel, 25 Karten mit Übungen, die aus dem ATP entnommen sind. Weitere Informationen:<br>www.aelter-werden-in-balance.de/bewegungspackung/

Hier finden Sie nähere Informationen zum Projekt www.aelter-werden-balance.de
